# Baicalein Mitigates Radiation-Induced Enteritis by Improving Endothelial Dysfunction

**DOI:** 10.3389/fphar.2019.00892

**Published:** 2019-08-16

**Authors:** Hyosun Jang, Janet Lee, Sunhoo Park, Joong Sun Kim, Sehwan Shim, Seung Bum Lee, Sung-Honn Han, Hyunwook Myung, Hyewon Kim, Won-Suk Jang, Sun-Joo Lee, Jae kyung Myung

**Affiliations:** ^1^Laboratory of Radiation Exposure & Therapeutics, National Radiation Emergency Medical Center, Korea Institute of Radiological and Medical Sciences, Seoul, South Korea; ^2^Department of Pathology, Korea Cancer Center Hospital, Korea Institute of Radiological and Medical Sciences, Seoul, South Korea; ^3^Herbal Medicine Resources Center, Korea Institute of Oriental Medicine, Daejeon, South Korea

**Keywords:** baicalein, intestinal inflammation, endothelial cells, adherent molecules, irradiation

## Abstract

**Background and Aims:** Radiation-induced intestinal injury occurred in application of radiotherapy for abdominal and pelvic cancers or in nuclear accidents. Radiation-induced enteritis may be considered an ideal model of gastrointestinal inflammation. The endothelium is a crucial component of inflammation, and the endothelial dysfunction following radiation exposure induces the intestinal proinflammatory response and progression of radiation enteritis. Baicalein (5,6,7-trihydroxyflavonoid) is a flavonoid from *Scutellaria baicalensis* used in oriental herbal medicine. Baicalein has been found to have multiple beneficial properties including antioxidant, anti-inflammatory, anti-allergic, and anti-cancer activities. Here, we investigated the therapeutic effects of baicalein on endothelial dysfunction in radiation-induced intestinal inflammation.

**Materials and Methods:** We performed histological analysis, bacterial translocation, and intestinal permeability assays and also assessed infiltration of leukocytes and inflammatory cytokine expression using a mouse model of radiation-induced enteritis. In addition, to investigate the effect of baicalein in endothelial dysfunction, we analyzed endothelial-derived adherent molecules in human umbilical vein endothelial cells (HUVECs) and irradiated intestinal tissue.

**Results:** Histological damage such as shortening of villi length and impaired intestinal crypt function was observed in the radiation-induced enteritis mouse model. Intestinal damage was attenuated in baicalein-treated groups with improvement of intestinal barrier function. Baicalein inhibited the expression of radiation-induced adherent molecules in HUVECs and intestine of irradiated mouse and decreased leukocyte infiltration in the radiation-induced enteritis.

**Conclusions:** Baicalein could accelerate crypt regeneration *via* recovery of endothelial damage. Therefore, baicalein has a therapeutic effect on radiation-induced intestinal inflammation by attenuating endothelial damage.

## Introduction

The intestine is one of the most radiosensitive organs in the body. Exposure to high doses of radiation, for example, following a nuclear accident or a dirty bomb explosion can cause severe intestinal damage with a high rate of mortality (Kavanagh et al., 2010). Similarly, the clinical application of radiation in the treatment of cancers in the abdominal and pelvic cavity may also lead to acute and/or chronic intestinal injury, which significantly reduces the quality of life as well as adds an extra burden to the cost of health care. Annually, 60–80% of patients who received abdominal or pelvic radiation therapy develop acute bowel toxicity. Acute bowel toxicity by irradiation is primarily a result of cell death in the proliferating crypt epithelium and an inflammatory reaction in the intestine. Intestinal crypt cell death induces a link of processes such as insufficient regeneration of the villi in epithelium, breakdown of the intestinal epithelial barrier, and mucosal inflammation. However, there are no effective treatment strategies available to mitigate radiation-induced intestinal injury (Greenberger, 2009; Citrin et al., 2010; Yu, 2013). Hauer-Jensen et al. recently proposed that radiation-induced enteritis is a useful model to explore mucosal inflammation, and the mechanisms related to radiation-induced intestinal inflammation could critically advance the understanding of gastrointestinal injury, such as inflammatory bowel disease (IBD) (Hauer‐Jensen et al., 2014; Toullec et al., 2017).

The endothelium has already been described as a crucial component involved in gastrointestinal disease, such as radiation-induced enteropathy and IBD (Paris et al., 2001; Cibor et al., 2016). Radiation induces many changes in endothelial cells, such as apoptosis, detachment from the basement membrane, increased endothelial permeability, interstitial fibrin deposition, and shifting of the thrombo-hemorrhagic balance toward coagulation. Endothelial dysfunction directly impacts the intestinal proinflammatory response following radiation exposure and progression of radiation enteritis (Korpela et al., 2014). In addition, endothelial cell damage leads to the loss and dysfunction of crypt stem cell clonogens (Maj et al., 2003). Previous experiments have indicated that the prevention of endothelial cell damage by growth factors (e.g., vascular endothelial growth factor and basic fibroblast growth factor) or plants extracts (e.g., coniferyl aldehyde) reduces intestinal crypt cell damage, inflammation, organ failure, and death in radiation-induced gastrointestinal toxicity (Okunieff et al., 1998; Jeong et al., 2015).

Baicalein (5,6,7-trihydroxyflavonoid) is one of the principal flavonoids extracted from the dry root of *Scutellaria baicalensis*. Baicalein has been found to have multiple beneficial properties, such as anti-oxidant, anti-bacterial, anti-inflammatory, anti-cancer, hypolipidemic, anti-atherogenic antithrombotic, and immunoregulatory effects. It is clinically used in the treatment of vasculitis and paralysis caused by cerebrovascular disorders (Kwak et al., 2014; Ge et al., 2017). Recent studies have discovered that baicalein potentially inhibited inflammation, endothelial damage, intracellular reactive oxygen species levels, and NF-kB activation in chemo agent-induced phlebitis (Ge et al., 2017). In addition, pre-treatment with baicalein reduces radiation-induced damage to the bone marrow cells (Gandhi, 2013). However, the therapeutic effects of baicalein in intestinal inflammation by radiation exposure remain unclear. In this study, we investigated whether baicalein attenuates acute radiation damage of the intestine to localize radiation, focusing on endothelial damage.

## Methods

### Mice

Specific pathogen-free (SPF) male C57BL/6 mice (7-week-old) were obtained from Harlan Laboratories (Indianapolis, IN, USA) and maintained under SPF conditions at the animal facility of the Korea Institute of Radiological and Medical Sciences (KIRAMS). All mice were housed in a temperature-controlled room with a 12-h light/dark cycle, and food and water were provided *ad libitum*. The mice were acclimated for 1 week before commencement of the experiments and were assigned to the following groups: 1) control (n = 22), 2) irradiation (IR, n = 22), and 3) irradiation with baicalein treatment (IR+Bai, n = 22). All animal experiments were performed in accordance with the guidelines of and were approved by the Institutional Animal Care and Use Committee of KIRAMS.

### Irradiation and Administration of Baicalein

Animals were anesthetized with an intraperitoneal injection of 85 mg/kg alfaxalone (Alfaxan^®^; Careside, Gyeonggi-do, Korea) and 10 mg/kg xylazine (Rompun^®^; Bayer Korea, Seoul, Korea). They were then irradiated with a single exposure to 13.5 Gy of whole abdominal irradiation at a dose rate of 2 Gy/min using an X-RAD 320 X-ray irradiator (Softex, Gyeonggi-do, Korea). After exposure, the animals were injected with an intraperitoneal dose of 10 mg/kg/day baicalein (Sigma-Aldrich, St. Louis, MO, USA) for 6 days.

### Histology and Immunostaining

Small intestine samples of mice were fixed with a 10% neutral buffered formalin solution, embedded in paraffin wax, and sectioned transversely to a thickness of 4 µm. The sections were then stained with hematoxylin and eosin (H&E) and Congo red. To perform immunohistochemical analysis, slides were subjected to antigen retrieval and then treated with 0.3% hydrogen peroxide in methyl alcohol for 20 min to block endogenous peroxidase activity. After three washes in phosphate-buffered saline (PBS), the sections were blocked with 10% normal goat serum (Vector ABC Elite kit; Vector Laboratories, Burlingame, CA, USA) and incubated with anti-mouse claudin 3 (CLDN3; Invitrogen, Carlsbad, CA, USA), anti-mouse zonula occludens-1 (ZO-1; Abcam, Cambridge, UK), anti-mouse P-selectin (Santacruz, Dallas, TX, USA), anti-mouse myeloperoxidase (MPO), anti-neutrophil elastase (Abcam), anti-mouse interleukin-6 (IL-6), and anti-mouse Ki-67 (Acris Antibodies GmbH, Herford, Germany) antibodies. After three washes in PBS, the sections were incubated with a horseradish peroxidase-conjugated secondary antibody (Dako, Carpinteria, CA, USA) for 60 min. The peroxidase reaction was developed using a diaminobenzidine substrate (Dako) prepared according to the manufacturer’s instructions, and the slides were counterstained with hematoxylin. For immunofluorescence of Cd4, rehydrated sections were used for antigen retrieval in pH 6.0 citrate buffer for 20 min. Sections were permeabilized in 0.3% TritonX-100 and blocked with 5% BSA at room temperature followed by incubation in anti-mouse CD4 antibody FITC conjugated (eBioscience, Vienna, Austria).

### Bacterial Translocation Assays

To evaluate the translocation of bacteria from the intestinal lumen to lymph nodes, mesenteric lymph nodes of mice were harvested under sterile conditions 6 days following IR. An aliquot of the mesenteric lymph node homogenates was plated onto MacConkey agar (BD, Franklin Lakes, NJ, USA) and incubated at 37°C for 18 h. Then, colonies were counted on all plates.

### Intestinal Permeability Assays

The animals were anesthetized, and a midline laparotomy was performed. A 5-cm segment of digital ileum was obstructed using bulldog clamps. An intraluminal injection of 12.5 mg fluorescein isothiocyanate (FITC)-dextran (4 kDa, Sigma, St Louis, MO) in 100 µl PBS was administered at 3 and 6 days following IR. At 30 min after intraluminal injection, blood was obtained *via* cardiac puncture and placed in serum-separating tubes. Blood was centrifuged at 1,000 × *g* for 15 min, and the serum was collected. The concentration of FITC-dextran in serum samples was analyzed using a fluorescence spectrophotometer (excitation: 485 nm, absorption: 528 nm).

### RNA Extraction, Reverse Transcription-Polymerase Chain Reaction (RT–PCR), and Real-Time PCR Quantification

Harvested mouse small intestine tissues were immediately snap-frozen and stored at −80°C until RNA extraction. Total RNA was isolated from the intestine tissues and human umbilical vein endothelial cells (HUVECs) using the TRIzol reagent (Invitrogen, Carlsbad, CA, USA). cDNA was synthesized using the AccuPower RT premix (Bioneer, Daejeon, Korea) according to the manufacturer’s protocol. Real-time RT-PCR was performed using a LightCycler 480 system (Roche, San Francisco, CA, USA). The primer sequences are provided in **Table 1**. The expression levels of each target gene, determined using the LightCycler 480 system software (Roche), were normalized to those of β-actin. Cycle threshold values were used to calculate relative mRNA expression using the 2^−ΔΔCt^ method.

**Table 1:** Real-time reverse transcriptase-polymerase chain reaction (RT–PCR) primer sequences.

Species	Primer	Forward (5′–3′)	Reverse (5′–3′)
Human	*P-SELECTIN*	TGAGCACTGCTTGAAGAAAAAGC	CACGTATTCACATTCTGGCCC
	*ICAM-1*	GGCCGGCCAGCTTATACAC	TAGACACTTGAGCTCGGGCA
	*VCAM-1*	TCAGATTGGAGACTCAGTCATGT	ACTCCTCACCTTCCCGCTC
	*GAPDH*	GGACTCATGACCACAGTCCATGCC	TCAGGGATGACCTTGCCCACAG
Mouse	*Claudin 3*	AAGCCGAATGGACAAAGAA	CTGGCAAGTAGCTGCAGTG
	*Zo-1*	AGGACACCAAAGCATGTGAG	GGCATTCCTGCTGGTTACA
	*P-selectin*	CAGCTAGCTAGCGAGAGGACATGGCTG GCTGCC	CCCAAGCTTGCCGCAATAGCTTCACAG GTTGGCAG
	*Icam-1*	GTGATGCTCAGGTATCCATCCA	CACAGTTCTCAAAGCACAGCG
	*Vcam-1*	GTTCCAGCGAGGGTCTACC	AACTCTTGGCAAACATTAGGTGT
	*Il-33*	CAATCAGGCGACGGTGTGGATGG	TCCGGAGGCGAGACGTCACC
	*Mmp-9*	GCCCTGGAACTCACACGACA	TTGGAAACTCACACGCCAGAAG
	*Il-6*	AGCCAGAGTCCTTCAGAGAG	GATGGTCTTGGTCCTTAGCC
	*Il-1β*	GCAACTGTTCCTGAACTCA	CTCGGAGCCTGTAGTGCAG
	*Cd-4*	TCCTAGCTGTCACTCAAGGGA	TCAGAGAACTTCCAGGTGAAGA
	*Il-13*	GCAGCATGGTATGGAGTGTG	TGGCGAAACAGTTGCTTTGT
	*β-actin*	TCCCTGGAGAAGAGCTATGA	CGATAAAGGAAGGCTGGAA

### ELISA

To determine the protein levels of inflammatory cytokines, intestinal tissue was tested using Quantikine ELISA kits for mouse IL-33 (R&D Systems, Minneapolis, MN, USA), mouse Myeloperoxidase-9 (MMP-9) (Cloud-Clone Corp., Houston, TX, USA), and IL-1β (BioLegend, San Diego, CA, USA).

### Cell Culture

HUVECs were purchased from Promocell and maintained in Endothelial Growth Medium-2 (EGM-2; Promocell GmbH, Heidelberg, Germany) in 0.1% gelatin-coated dishes. Cells were irradiated with 10 Gy of irradiation using a ^137^Cs γ-ray source (Atomic Energy of Canada, Chalk River, ON, Canada) at a dose rate of 3.81 Gy/min and then treated with baicalein (Sigma–Aldrich, St. Louis, MO, USA) within 1 h. After 6 h of incubation, the cells were used for experiments.

### Crypt Isolation and Isolated Crypt Counting

Isolation of small intestinal crypts from mice in each condition was conducted as described previously (Han et al., 2017). Briefly, the small intestines from Con, IR, and IR+Bai mice at 6 days after IR were longitudinally opened and then cut into approximately 5-mm pieces. The intestinal fragments were washed three times with cold PBS and then incubated with 2 mM EDTA in PBS for 15 min at 37°C. After incubation, the supernatant containing villi was discarded and replaced with cold PBS. The solution with tissue fragments was vigorously hand-shaken for 1 min. This procedure was repeated three times. After obtaining the isolated crypts by centrifugation, the crypts resuspended in 2% D-sorbitol (Sigma, St. Louis, MO, USA) in PBS were passed through a 70-µm cell strainer (BD Biosciences, Heidelberg, Germany). After centrifugation, the pellet was resuspended in 10 ml of a basic medium (advanced Dulbecco’s modified Eagle’s medium/F12, 0.1% bovine serum albumin, 2 mM l-glutamine, 10 mM HEPES, 100 mg/ml streptomycin, 100 U/ml penicillin, 1 mM N-acetylcysteine, 1% B27, and N2 supplement). Images of isolated crypts were acquired using a microscope (Olympus, Shinjuku, Tokyo, Japan).

### Primary Culture of Intestinal Organoids

One hundred crypts from isolated Con, IR, and IR+Bai group were embedded within 50 µl Matrigel (BD Biosciences) in each well of a 24-well cell culture plate. After incubation for 30 min at 37°C, 500 µl of epidermal growth factor (EGF)/Noggin/R-spondin-1 (ENR) medium was added. Crypts were cultured at 37°C in an atmosphere containing 5% CO_2_ for 7 days. During culture, ENR medium was changed every 2–3 days. The ENR medium contained basic medium plus 50 ng/ml murine EGF (Invitrogen, Carlsbad, CA, USA), 100 ng/ml murine Noggin (Peprotech, Hamburg, Germany), and 500 ng/ml human R-spondin-1 (R&D Systems, Minneapolis, MN, USA). For quantitative analysis of organoid formation, the number of organoids with two or more budding structures in each group was counted under bright-field microscopy at 7 days. Experiments were performed in triplicate.

### Statistical Analysis

All quantitative data are expressed as mean ± standard error of the mean. Statistical significance of differences was evaluated by performing a one-way analysis of variance (ANOVA) with a Tukey’s multiple comparison test. A *P*-value of <0.05 was considered statistically significant.

## Results

### Baicalein Alleviates Radiation-Induced Intestinal Injury

**Figure 1:**
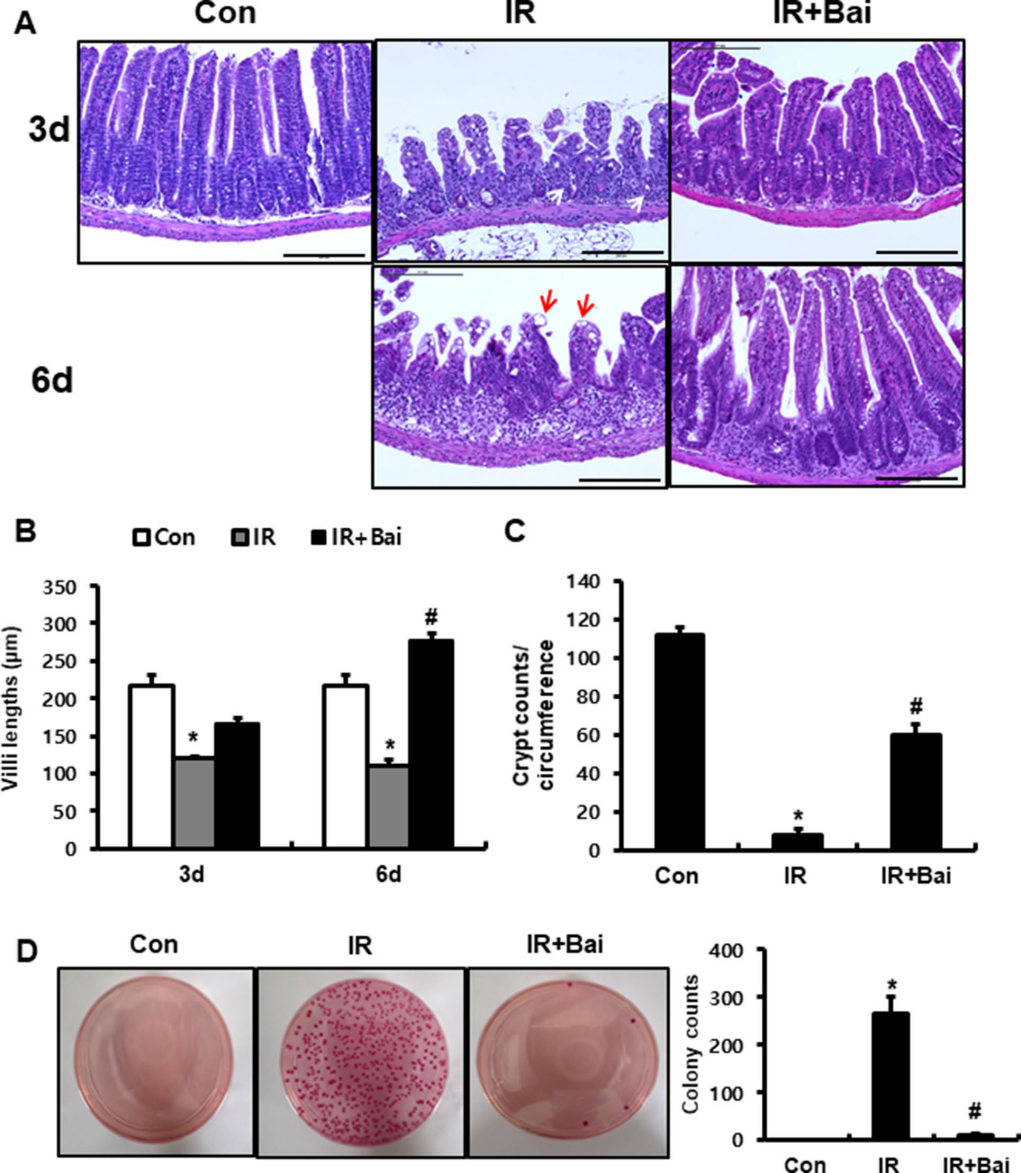
Baicalein alleviates radiation-induced intestinal injury and inhibits bacterial translocation. **(A)** Hematoxylin–eosin (H&E)-stained small intestine tissues harvested from control (Con), irradiated (IR), and baicalein-treated IR (IR+Bai) mice 3 and 6 days after application of 13.5 Gy of abdominal irradiation. Bar = 200 μm. **(B)** Villi lengths in the small intestine from Con, IR, and IR+Bai mice at 3 and 6 days after irradiation (50 villi for each group). **(C)** Crypt numbers of circumference in the small intestine from Con, IR, and IR+Bai mice at 6 days after irradiation (15 crypt for each group). **(D)** The number of colonies from mesenteric lymph nodes tissue of Con, IR, and IR+Bai mice. White arrow indicates crypt abscess, and red arrow indicates epithelial cell vacuolization. Data are presented as the mean ± standard error of the mean; n = 5 mice per group. *P < 0.05 compared to the control; *^#^* *P* < 0.05 compared to the IR group.

To investigate the effects of baicalein on radiation-induced intestinal injury, we performed localized irradiation on the whole abdomen of mice of 13.5 Gy using an Xrad-320. First, we analyzed the histological alteration to identify the therapeutic effects of baicalein on acute radiation-induced intestinal injury. After irradiation, remarkable crypt destruction with abscess (white arrow), villi shortening, epithelial cell vacuolization (red arrow), and inflammatory cell infiltration and edematous change in mucosa and submucosa were observed in the intestine at 3 and 6 days (**Figures 1A–C**). Villi length at 3 and 6 days and crypt numbers at 6 days were significantly increased in baicalein-treated irradiated mice than those in the irradiated group (**Figures 1B, C**). Bacterial translocation to the lymph nodes indicates epithelial defect of the intestine, and we determined that bacterial translocation to mesenteric lymph nodes significantly increased in irradiated mice than in control mice (**Figure 1D**). On the other hand, bacterial translocation to the mesenteric lymph nodes decreased in baicalein-treated irradiated mice (**Figure 1D**). Therefore, baicalein treatment alleviated radiation-induced intestinal injury with inhibition of bacterial translocation.

### Baicalein Improves Intestinal Barrier Function With Upregulated Tight Junctional Molecules

**Figure 2:**
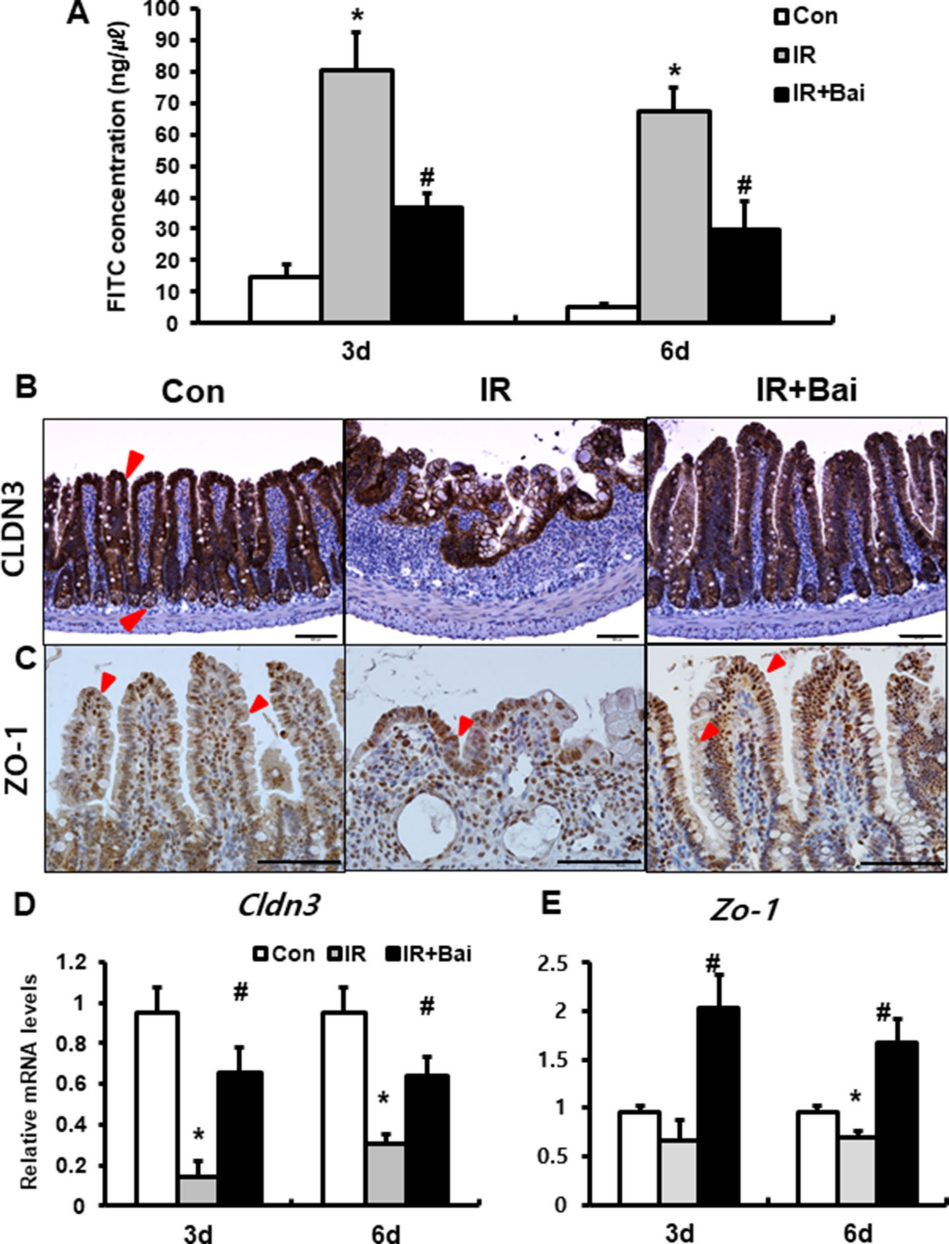
Baicalein recoveries intestinal barrier damage by radiation exposure. **(A)** FITC-dextran absorption assay of control (Con), irradiated (IR), and baicalein-treated IR (IR+Bai) mice. Immunohistochemistry of **(B)** claudin 3 (CLDN3) and **(C)** zonula occludens-1 (ZO-1) and mRNA expression of **(D)** *Cldn3* and **(E)** *Zo-1* in the intestinal tissue of Con, IR, and IR+Bai mice. Red arrow indicates each positive cell. Bar = 100 μm. Data are presented as the mean ± standard error of the mean; n = 5 mice per group. *P < 0.05 compared to the control; *^#^* *P* < 0.05 compared to the IR group.

To identify that baicalein attenuates barrier function of the small intestine, we assessed FITC-dextran absorption assays and expression of tight junctional molecule expression. The concentration of FITC in the serum was significantly increased in the irradiated group compared to that in the control mice (**Figure 2A**). Baicalein treatment decreased FITC levels compared to those in the irradiated group (**Figure 2A**). Tight junctions, which are highly specialized intercellular junctions, are responsible for epithelial barrier functions in the gastrointestinal tract (Turner, 2009). Especially, CLDN3 and ZO-1 sensitively respond to radiation exposure and affect intestinal barrier function (Shim et al., 2015; Shukla et al., 2016). Protein expressions of CLDN3 and ZO-1 were upregulated in the irradiated intestine with baicalein treatment (**Figures 2B, C**). In addition, we also identified that mRNA levels of *Cldn3 and Zo-1* were significantly increased in the baicalein-treated irradiated group compared to the levels in the only irradiated group (**Figures 2D, E**). These results suggested that baicalein not only attenuated radiation-induced intestinal injury but also improved intestinal barrier dysfunction *via* increased tight junction expression.

### Baicalein Inhibits Leukocyte Infiltration by Downregulating Endothelial Adhesion Molecules

Endothelial dysfunction is also a key determinant of both acute and chronic organ damage associated with irradiation of the gastrointestinal tract (Paris et al., 2001). Damaged endothelial cells can induce inflammatory cascade by expressing inflammatory cytokines and adhesion molecules, such as intercellular adhesion molecule-1 (ICAM-1), vascular cell adhesion molecule-1 (VCAM-1), E-selectin, and P-selectin. These adhesion molecules sustain leukocyte adherence to vascular endothelium and aid subsequent transendothelial migration into the inflamed tissue (Quarmby et al., 1999).

**Figure 3:**
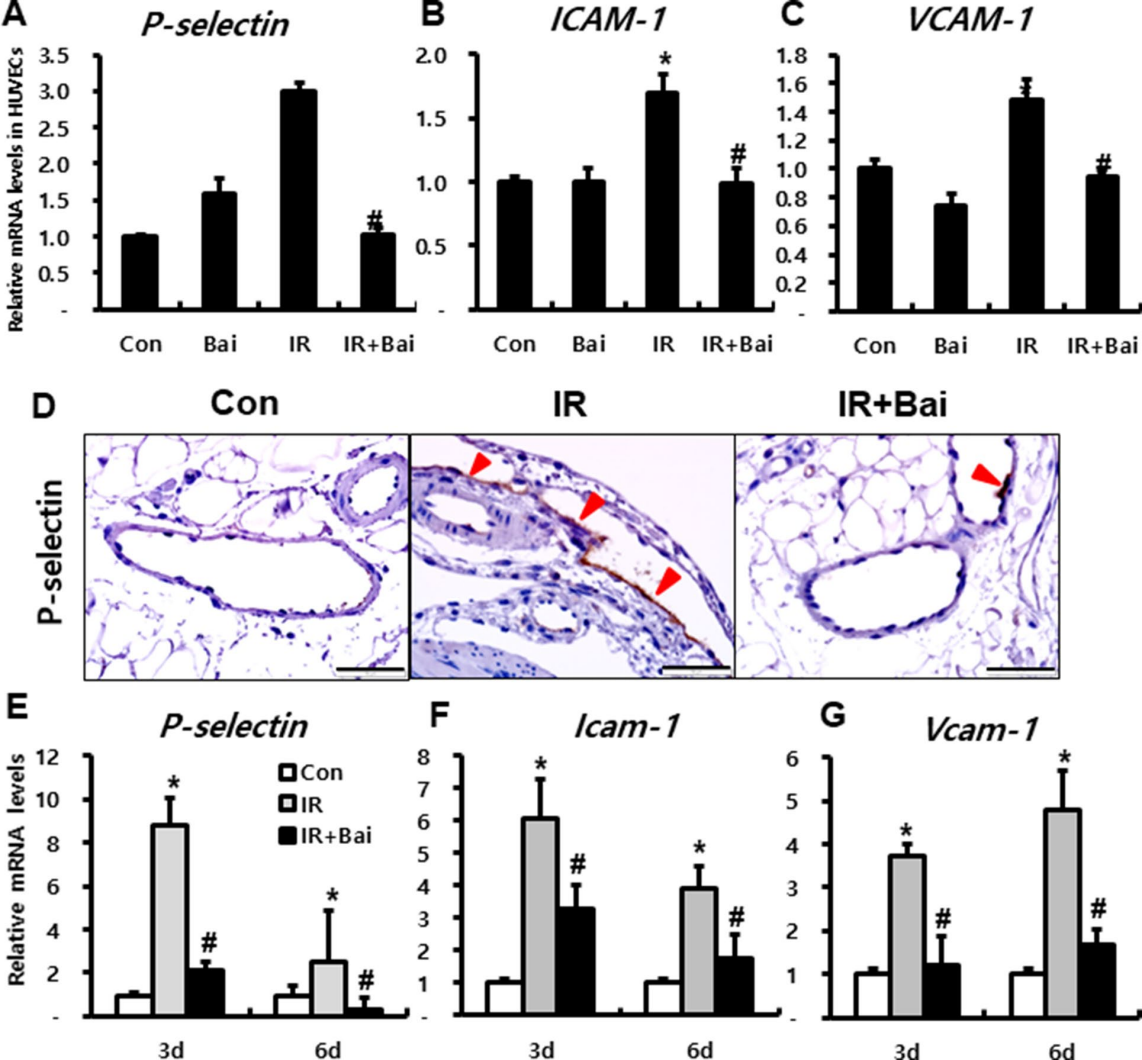
Baicalein attenuates endothelial dysfunction in radiation-induced intestinal injury. mRNA levels of **(A)** *P-selectin*, **(B)** *ICAM-1*, and **(C)** *VCAM-1* in control (Con), baicalein treated (Bai), irradiated (IR), and baicalein-treated IR (IR+Bai) HUVECs, as determined by real-time RT-PCR. n = 3 per group. **(D)** Immunohistochemical analysis of P-selectin and mRNA levels of **(E)** *P-selectin*, **(F)** *Icam-1*, *and* **(G)** *Vcam-1* in the intestine of Con, IR, and IR+Bai mice. Arrowhead indicates P-selectin-positive endothelial cell. Bar = 100 μm. Data are presented as the mean ± standard error of the mean; n = 5 mice for each group. *P < 0.05 compared to the control; *^#^* *P* < 0.05 compared to the IR group.

To investigate the effects of baicalein on expression of adherent molecules in endothelial cells after irradiation, we performed *in vitro* experiment using HUVECs. mRNA expression of endothelial-derived adherent molecules, such as *P-selectin*, *ICAM-1*, and *VCAM-1*, markedly increased in the irradiated HUVECs compared to that in the control group (**Figures 3A–C**). Moreover, baicalein attenuated the expression of *P-Selectin*, *ICAM-1*, and *VCAM-1* compared to that in irradiated HUVECs (**Figures 3A–C**).

In the intestinal tissue of irradiated mouse, we also identified that P-selectin was strongly expressed on the endothelial cells of mesenteric lesions in IR mice (**Figure 3D**). Furthermore, baicalein attenuated immunoreactivity of P-selectin compared to that in the IR mice (**Figure 3D**). The mRNA levels of adherent molecules such as *P-selectin*, *Icam-1*, and *Vcam-1* significantly increased in the irradiated intestine compared to those in the control mice (**Figures 3E–G**). Baicalein inhibited the expression of these molecules in the irradiated intestine (**Figures 3E–G**).

**Figure 4:**
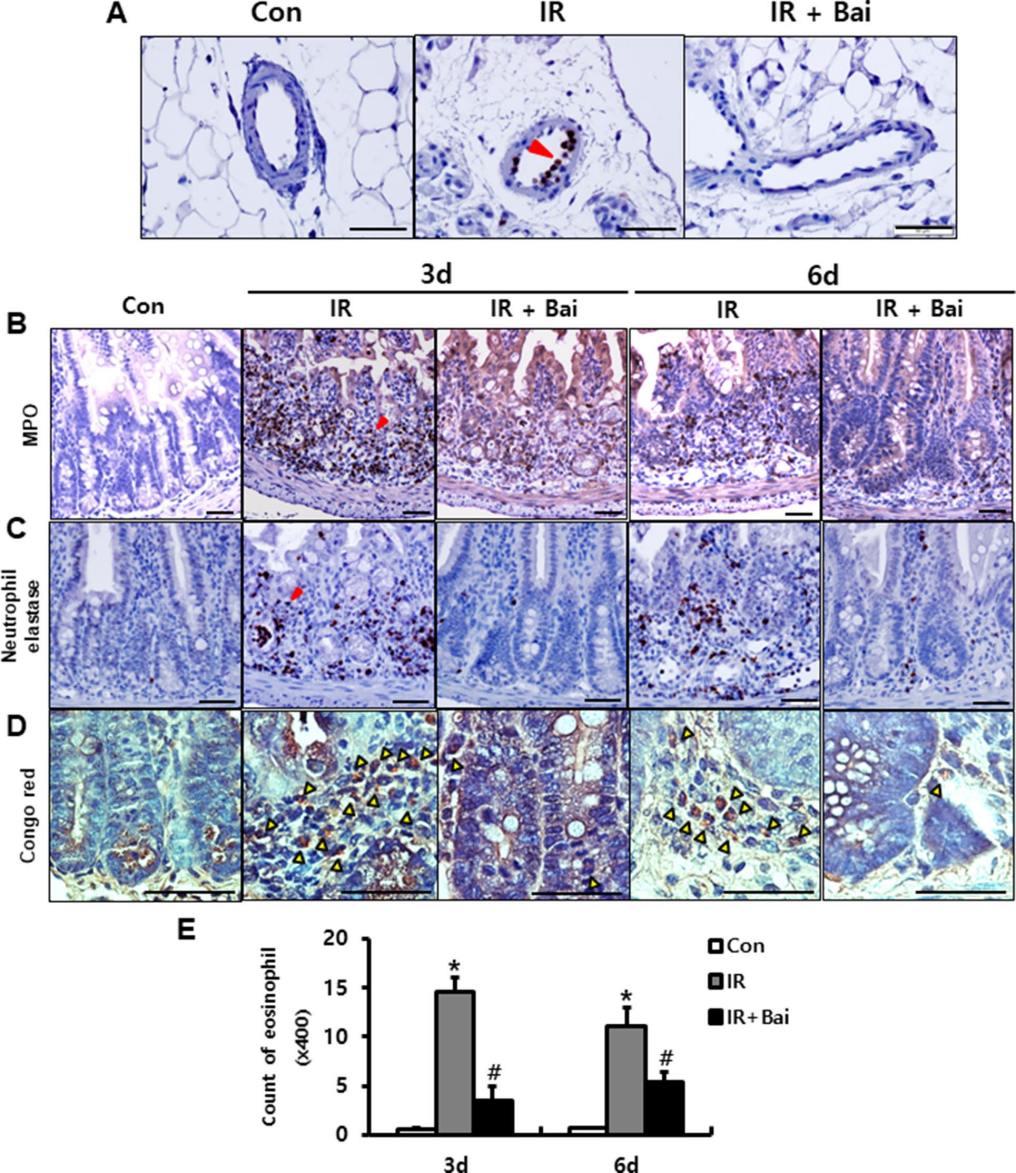
Baicalein inhibits leukocyte infiltration in radiation-induced enteritis. **(A)** Attachment of elastase-positive neutrophil in the mesenteric vessels in the intestine of control (Con), irradiated (IR), and baicalein-treated IR (IR+Bai) mice at 6 days after radiation exposure. Immunohistochemistry of **(B)** myeloperoxidase (Mpo) and **(C)** neutrophil elastase for neutrophil detection, and **(D)** Congo red stain for eosinophil detection in the intestine of Con, IR, and IR+Bai mice. Red arrowhead indicates Mpo- and elastase-positive neutrophils. Yellow arrowhead indicates eosinophil. **(E)** Eosinophil counting in the intestine of Con, IR, and IR+Bai mice. Bar = 100 μm. Data are presented as the mean ± standard error of the mean; n = 5 mice for each group. *P < 0.05 compared to the control; *^#^* *P* < 0.05 compared to the IR group.

Leukocyte infiltration of injured tissues is a hallmark of inflammation. During inflammatory processes, leukocytes roll on endothelial cells, arresting and transmigrating between them when they are sequestered from the circulation (Lawrence and Springer, 1991). Neutrophils, one of the leukocytes, undergo respiratory burst and contribute to the progression of the inflammatory response in complex ways (Williams et al., 2011). We also identified the elastase-positive neutrophil detected in the mesenteric vessels of irradiated intestine (**Figure 4A**). Baicalein inhibited the attachment of elastase-positive neutrophils in the mesenteric endothelial cells (**Figure 4A**). Additionally, we assessed the infiltration of neutrophils in the intestinal tissue using neutrophil markers, which are neutrophil elastase and MPO. Irradiated intestine showed increased infiltration of neutrophils in the mucosal and submucosal layers at 3 and 6 days after radiation exposure (**Figures 4B, C**). Baicalein decreased the neutrophil infiltration in the intestine compared to that in the irradiated group at 3 and 6 days (**Figures 4B, C**). Eosinophils play an important role in intestinal inflammation and radiation-induced intestinal fibrosis by releasing cationic proteins, cytokines [e.g., interleukin (IL)-6, IL-10, and IL-13], and chemokines [e.g., C-C motif ligand (CCL)3, CCL5, and CCL17] (Radnai et al., 2016; Takemura et al., 2018). A few eosinophils were seen in the submucosal layer of the healthy intestine (**Figures 4D, E**). In addition, irradiated intestine showed significantly increased number of eosinophils in the submucosal layer compared to that in control mice (**Figures 4D, E**). Baicalein decreased eosinophil numbers in the irradiated intestine (**Figures 4D, E**). These results suggested that baicalein attenuated infiltration of neutrophils and eosinophils by inhibiting expression of endothelial-derived adhesion molecules related with adhesion of neutrophils and eosinophil to endothelial cells.

### Baicalein Inhibits Radiation-Induced Intestinal Inflammation Response

**Figure 5:**
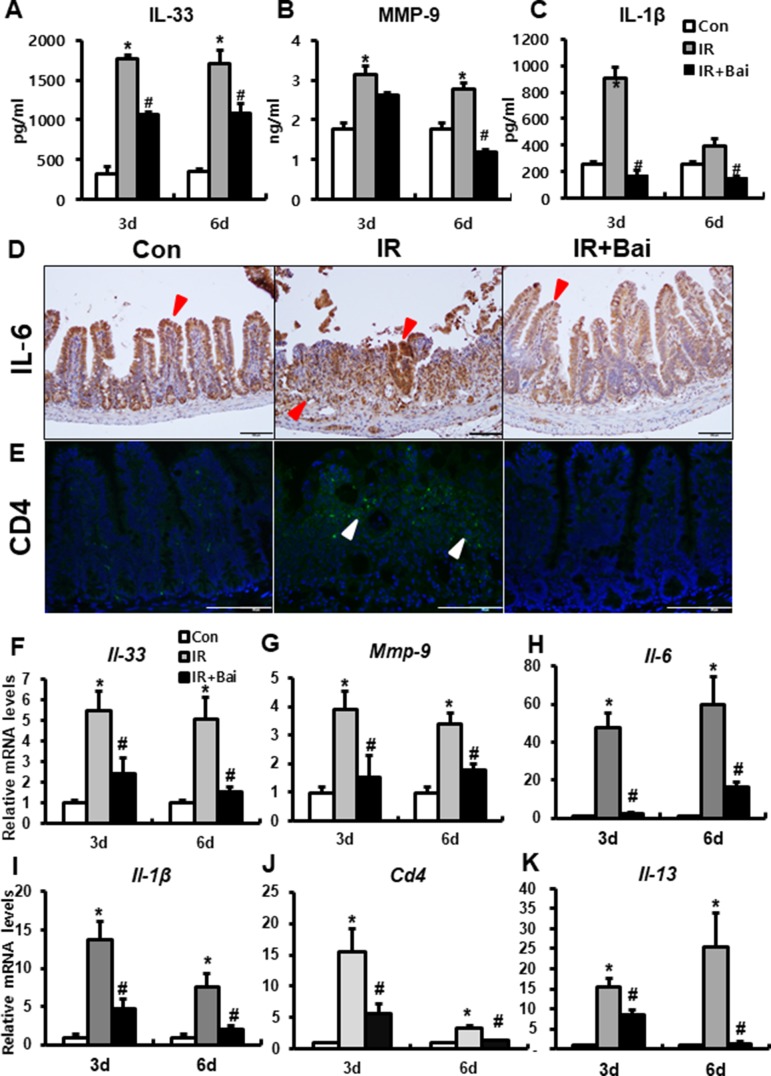
Baicalein inhibits the inflammatory response during radiation-induced enteropathy. Protein expression of **(A)** interleukin (IL)-33, **(B)** matrix metalloproteinase-9 (MMP-9), and **(C)** IL-1β in the intestine of control (Con), irradiated (IR), and baicalein-treated IR (IR+Bai) mice. Immunostaining of **(D)** IL-6 and **(E)** CD4 in the intestine of Con, IR, and IR+Bai mice. mRNA levels of **(F)** *Il-33*, **(G)** *Mmp-9*, **(H)** *Il-6*, **(I)** *Il-1*β, **(J)** *Cd4*, and **(K)** *Il-13* in the intestine of Con, IR, and IR+Bai mice. Red arrow indicates IL-6 positive cell and white arrow indicated CD4 positive cell. Bar = 100 μm. Data are presented as the mean ± standard error of the mean; n = 5 mice for each group. *P < 0.05 compared to the control; *^#^* *P* < 0.05 compared to the IR group.

A previous study has shown that irradiation of the intestine increased the (pro-)inflammatory cytokines, such as IL-33, IL-6, IL-1β, and MMP-9 (Jang et al., 2018). We also identified that the protein and mRNA expression of inflammatory cytokines and chemokines, such as IL-33, IL-6, IL-1β, and MMP-9, markedly increased in the irradiated intestine compared to that in the control group (**Figures 5A–D and F–I**). Radiation-induced inflammation characterizes T helper 2 (Th2)-type inflammation, which is increased Th2 cell infiltration and increased IL-4, IL-5, and IL-13 (Grémy et al., 2008; Gao et al., 2018). In our data, we also assessed the expression of CD4 and *Il-13* in the irradiated intestine. The IR group showed a lot of CD4-positive cells in mucosal layer and increased mRNA levels of *Cd4* and *Il-13* compared to the control group (**Figures 5E, J, K**). Baicalein attenuated the level of IL-33, MMP-9, IL-6, IL-1β, CD4, and *Il-13* in the irradiated intestine (**Figures 5A–K**). These results suggested that baicalein inhibited the inflammation response following radiation exposure.

### Baicalein Attenuates Radiation-Induced Crypt Damage

**Figure 6:**
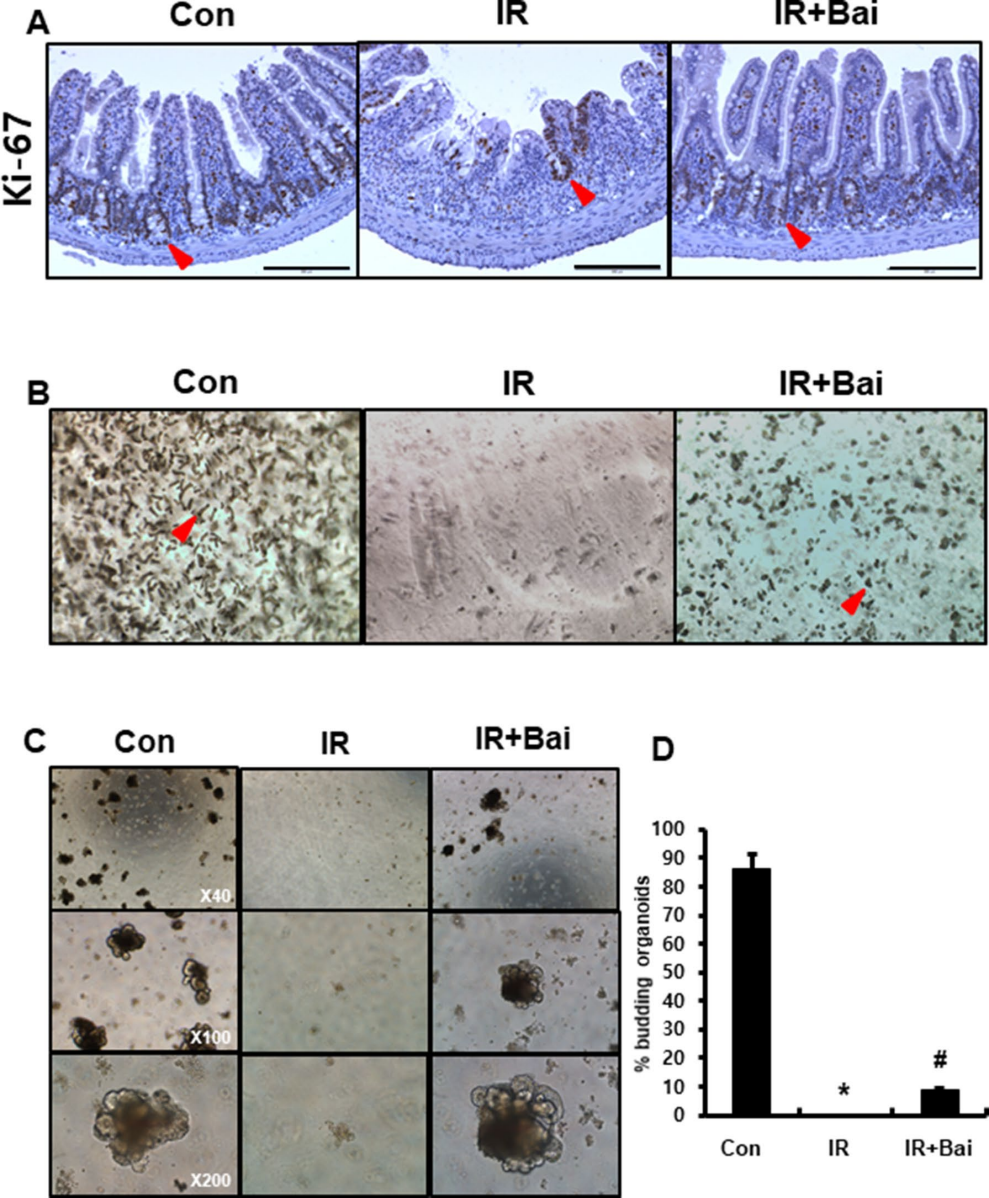
Baicalein improves crypt regeneration in radiation-induced enteritis. **(A)** Immunohistochemistry of Ki-67 in the intestine of control (Con), irradiated (IR), and baicalein-treated IR (IR+Bai) mice at 6 days after irradiation. Red arrow indicates Ki-67 positive cells. **(B)** Representative morphology of isolated intestinal crypts in Con, IR, and IR+Bai mice at 6 days after irradiation. Red arrow indicates crypts. **(C)** Enteroid formation assay cultured *in vitro* for 7 days and **(D)** percentage of budding organoids in Con, IR, and IR+Bai groups. Data are presented as the mean ± standard error of the mean; n = 3 samples for each group. *P < 0.05 compared to the control; *^#^* *P* < 0.05 compared to the IR group.

To evaluate the proliferative activity and repopulation ability in baicalein-treated irradiated mice, we performed immunohistochemistry for the proliferation marker Ki-67 and enteroid formation assay on intestinal tissues. The intestines of the irradiated group exhibited few Ki-67-positive cells, whereas those of the baicalein-treated irradiated group showed increased Ki-67 positive cells in the crypts (**Figure 6A**). Isolated intestinal crypts or intestinal stem cells can be grown *in vitro* to form enteroids that comprise all the differentiated intestinal cell types found in the intestinal crypts (Sato and Clevers, 2013). This primary cell culture model can be used to simulate the physiology of the intestinal epithelium. We performed isolation of intestinal crypts of mice that were exposed to 13.5 Gy of abdominal irradiation and cultured *in vitro*. When collected 6 days after radiation, baicalein-treated irradiated group displayed an increased number of crypt isolation compared to the irradiated groups (**Figure 6B**). When the same number of crypts from each group was cultured in ENR medium, the intestinal crypts isolated from the baicalein-treated irradiated group showed more and larger enteroids than those from irradiated group (**Figures 6C, D**). These results suggested that baicalein attenuated radiation-induced crypt damage.

## Discussion

Although many efforts have been made to minimize radiation toxicity in healthy tissues (for example, fractionated low-dose radiation) during radiotherapy, more than half of the patients suffer from intestinal injury, including diarrhea, bleeding, stiffness, and fistula due to acute damage of crypt epithelium (Haydont and Vozenin‐Brotons, 2007; Hauer-Jensen et al., 2014; Takemura et al., 2018). Moreover, as there are increasing concerns regarding radiation exposure either following an accidental leakage or from terrorist activity, research has focused on developing medical countermeasures against radiation-induced gastrointestinal injury.

In this study, we investigated the effects of baicalein on radiation-induced enteritis with a localized abdominal irradiation model because baicalein possessed anti-inflammatory activity, which is effective in treating intestinal inflammatory disorders (Bae et al., 2016). Baicalein, a natural flavone, has multiple beneficial properties including anti-apoptotic, anti-inflammatory, anti-allergic, and anti-cancer (Lee et al., 2011; Mabalirajan et al., 2013; Guo et al., 2015). Treatment with baicalein has been reported to attenuate inflammatory responses, such as endothelium intimal hyperplasia, radiation-induced nephritis, and IBD model (Peng et al., 2008; Kim et al., 2014).

Radiation exposure induces pathophysiological damage including crypt destruction, epithelial cell vacuolization, villi shortening, and inflammatory cell infiltration in the intestine. In addition, epithelial cell damage induces intestinal barrier dysfunction resulting in bacterial translocation from the intestinal lumen to the blood, which can progress to inflammation and endotoxemia (Fukata et al., 2005; Jang et al., 2018). Moreover, this destruction of the barrier function leads to intestinal disorders such as leaky gut syndrome, IBD, and food allergies. In this study, we showed that radiation exposure induced epithelial damage with crypt destruction and increased intestinal permeability with decreased tight junction expression. Baicalein attenuated histological damages such as villi length and crypt numbers and aided in the recovery of intestinal barrier function.

Endothelium regulates the migration of leukocytes into the interstitial space by controlling adhesive molecule expression and producing chemokines, cytokines, and growth factors (D’Alessio et al., 2013). Therefore, endothelial cell dysfunction is already believed to be a key modulator of intestinal inflammation in radiation bowel toxicity and IBD (Paris et al., 2001; Cibor et al., 2016). Because vasculature permeability is increased within hours after radiation exposure, microvascular endothelial cells are damaged in early time following irradiation. Activated endothelial cells secrete chemoattractants and express adhesion molecules such as P- and E-selectin, ICAM-1, and VCAM-1. P and E-selectin mediate leukocyte rolling and ICAM-1 and VCAM-1 are the key molecules mediating leukocyte arrest in inflammatory response (Quarmby et al., 1999). Microvascular expression of ICAM-1 and VCAM-1 is upregulated in patients with IBD (Hatoum et al., 2003). Hallahan and Virudachalam demonstrated that irradiated endothelial cells increase ICAM-1 expression and an ICAM-1 antibody treatment or ICAM-1 deficiency model reduces radiation-induced inflammation of the lung (Hallahan and Virudachalam, 1997). Pravastatin mitigated radiation-induced skin damage through diminished endothelial activation with decreased ICAM-1 expression and inhibition of neutrophil recruitment (Holler et al., 2009). We also identified that radiation exposure upregulated the expression of P-selectin, ICAM-1, and VCAM-1 in HUVECs and intestinal tissue. Especially, P-selectin was strongly expressed in the mesenteric endothelial surface and resulted in neutrophil adherence in the mesenteric vessels of the irradiated intestine. On the other hand, baicalein alleviated the expression of these adherent molecules and decreased neutrophil attachment in the irradiated mesenteric vessel.

Leukocyte infiltration in inflamed tissue is one of the important components of progress of inflammatory processes. Expression of adhesion molecules on the surface of endothelial cells sustains leukocyte adherence to vascular endothelium and aids in subsequent transendothelial migration into the inflamed tissue. Principally, neutrophils bind the adhesion molecules and undergo transendothelial diapedesis into the damaged tissue, where they can undergo respiratory burst and contribute to the progression of the inflammatory response in complex ways (Williams et al., 2011). Eosinophils are also proinflammatory leukocytes that constitute a small percentage of circulating blood cells (Al‐Haddad and Riddell, 2005). Eosinophils partly contribute to the inflammatory process through release of various toxicity protein and cytokines that derived in concert with other inflammatory and immune cells. Patients and animal models of IBD display increased eosinophils in inflammatory lesion of the intestinal tissue. Depletion of eosinophils also attenuates the inflammatory response (Al‐Haddad and Riddell, 2005; Radnai et al., 2016). In our data, neutrophil infiltration appeared in the irradiated intestine with increased expression of adherent molecules compared to that seen in the control group. We also identified significant increase of eosinophils in the irradiated intestine. Baicalein inhibited neutrophil and eosinophil infiltration with decreased adherent molecule expression in radiation-induced intestinal injury.

As IL-33 is an epithelial- and endothelial-derived proinflammatory cytokine, it has a direct effect on eosinophil infiltration and function in the gut mucosa that potentially leads to intestinal inflammation (De Salvo et al., 2016). IL-33 triggers the production of IL-13 from the eosinophils, and this is important in Th2-type immune response (Rankin et al., 2010). Overexpression of IL-33 has been reported in the intestinal mucosa of patients with IBD (Nunes et al., 2014). We showed that *Il-33* was significantly increased in irradiated intestinal tissue with eosinophil infiltration and upregulation of *Il-13* and *Cd4* expression. These results suggest that radiation-induced enteritis is characterized by Th2-type inflammation corresponding to the previous reports (Grémy et al., 2008; Gao et al., 2018).

Radiation-induced intestinal injury displays inflammation reaction with increased inflammatory cytokines and chemokines such as IL-6, IL-1β, and MMP-9 (Jang et al., 2018). IL-6 is an important acute inflammatory phase mediator (Nishimoto and Kishimoto, 2006). IL-1β is produced immediately following tissue irradiation in epithelial cells and endothelial cells, and contributes to the inflammatory reaction (Liu et al., 2006). MMP-9 is the most abundantly expressed protease in inflamed tissues, and neutrophils were proposed as the most likely cellular source of this enzyme (Baugh et al., 1999). In our data, the expression of IL-6, IL-1β, and MMP-9 increased in the intestinal tissues of the irradiated group and the result suggested that intestinal inflammation is accompanied by endothelial dysfunction in radiation-induced intestinal injury. Baicalein displayed anti-inflammatory effects by attenuating endothelial dysfunction and the inflammatory mediators, such as IL-33, IL-6, IL-1β, MMP-9, and IL-13, in radiation enteropathy.

Endothelial dysfunction by radiation exposure directly impacts the intestinal proinflammatory response and progression of radiation enteritis (Korpela and Liu, 2014), as well as leads to the loss and dysfunction of crypt stem cell clonogens (Maj et al., 2003). Researchers suggest that the prevention of endothelial cell damage attenuates crypt cell damage in radiation-induced gastrointestinal syndrome (Okunieff et al., 1998; Jeong et al., 2015). We also observed that radiation exposure decreased crypt numbers, crypt proliferative property, and regeneration of the crypts in the intestine using *in vivo* and *ex vivo* systems. Baicalein accelerated the regenerative property of the crypts with recovery of endothelial dysfunction.

## Conclusion

In this study, we demonstrated that baicalein significantly alleviated radiation-induced intestinal injury and inhibited the expression of adherent molecules on the endothelial cells. The decrease of adherent molecules by baicalein results in regulation of inflammation, intestinal barrier recovery, and crypt regeneration. Our data support the further investigation of baicalein as a potential therapeutic agent for intestinal inflammation.

## Data Availability

The raw data supporting the conclusions of this manuscript will be made available by the authors, without undue reservation, to any qualified researcher.

## Ethics Statement

All animal experiments were performed in accordance with the guidelines of and were approved by the Institutional Animal Care and Use Committee of KIRAMS.

## Author Contributions

Conceived and designed the experiments: HJ, JL, SP, JSK, JKM. Performed the experiments: HJ, JL, SS, SBL, S-HH, HM, HK, W-SJ, S-JL. Analyzed the data: HJ, JL, HK. Contributed reagents/material/analysis tools: SP, JSK, JKM. Wrote the paper: HJ, JL, JKM.

## Funding

This study was supported by a grant from the Korea Institute of Radiological and Medical Sciences (KIRAMS), funded by the Ministry of Science and ICT (MSIT), Republic of Korea (No. 50535-2019).

## Conflict of Interest Statement

The authors declare that the research was conducted in the absence of any commercial or financial relationships that could be construed as a potential conflict of interest.

## Abbreviations

IBD, inflammatory bowel disease; SPF, specific pathogen-free; KIRAMS, Korea Institute of Radiological and Medical Sciences; IR, irradiation; H&E, hematoxylin and eosin; PBS, phosphate-buffered saline; MPO, myeloperoxidase; cldn3, claudin 3; FITC, fluorescein isothiocyanate; RT-PCR, reverse transcription-polymerase chain reaction; HUVECs, human umbilical vein endothelial cells; EGM-2, Endothelial Growth Medium-2; EGF, epidermal growth factor; ENG, EGF/Noggin/R-spondin-1; ZO-1, zonula occludens-1; ICAM-1, intercellular adhesion molecule-1; VCAM-1, vascular cell adhesion molecule-1; IL, interleukin; CCL, C-C motif ligand; MMP-9, matrix metalloproteinase-9; T helper 2, Th2; Con, control; IR, irradiation; IR+Bai, Baicalein-treated IR.

## References

[B1] Al-Haddad S. Riddell R. H. (2005). The role of eosinophils in inflammatory bowel disease. Gut 54, 1674–1675. 10.1136/gut.2005.072595 16284283 PMC1774805

[B2] Bae M. J. Shin H. S. See H. J. Jung S. Y. Kwon D. A. Shon D. H. (2016). Baicalein induces CD4(+)Foxp3(+) T cells and enhances intestinal barrier function in a mouse model of food allergy. Sci. Rep. 6, 32225. 10.1038/srep32225 27561877 PMC4999817

[B3] Baugh M. D. Perry M. J. Hollander A. P. Davies D. R. Cross S. S. Lobo A. J. (1999). Matrix metalloproteinase levels are elevated in inflammatory bowel disease. Gastroenterology. 117, 814–822. 10.1016/S0016-5085(99)70339-2 10500063

[B4] Cibor D. Domagala-Rodacka R. Rodacki T. Jurczyszyn A. Mach T. Owczarek D. (2016). Endothelial dysfunction in inflammatory bowel diseases: pathogenesis, assessment and implications. World J. Gastroenterol. 22, 1067–1077. 10.3748/wjg.v22.i3.1067 26811647 PMC4716020

[B5] Citrin D. Cotrim A. P. Hyodo F. Baum B. J. Krishna M. C. Mitchell J. B. (2010). Radioprotectors and mitigators of radiation-induced normal tissue injury. Oncologist 15, 360–371. 10.1634/theoncologist.2009-S104 20413641 PMC3076305

[B6] D’Alessio S. Tacconi C. Fiocchi C. Danese S. (2013). Advances in therapeutic interventions targeting the vascular and lymphatic endothelium in inflammatory bowel disease. Curr. Opin. Gastroenterol. 29, 608–613. 10.1097/MOG.0b013e328365d37c 24100721

[B7] De Salvo C. Wang X. M. Pastorelli L. Mattioli B. Omenetti S. Buela K. A. (2016). IL-33 drives eosinophil infiltration and pathogenic type 2 helper T-cell immune responses leading to chronic experimental ileitis. Am. J. Pathol. 186, 885–898. 10.1016/j.ajpath.2015.11.028 26908008 PMC5807926

[B8] Fukata M. Michelsen K. S. Eri R. Thomas L. S. Hu B. Lukasek K. (2005). Toll-like receptor-4 is required for intestinal response to epithelial injury and limiting bacterial translocation in a murine model of acute colitis. Am. J. Physiol. Gastrointest. Liver Physiol. 288, 1055–1065. 10.1152/ajpgi.00328.2004 15826931

[B9] Gandhi N. M. (2013). Baicalein protects mice against radiation-induced DNA damages and genotoxicity. Mol. Cell. Biochem. 379, 277–281. 10.1007/s11010-013-1649-z 23606056

[B10] Gao H. Dong Z. Gong X. Dong J. Zhang Y. Wei W. (2018). Effects of various radiation doses on induced T-helper cell differentiation and related cytokine secretion. J. Radiat. Res. 59, 395–403. 10.1093/jrr/rry011 29554285 PMC6054226

[B11] Ge G. F. Shi W. W. Yu C. H. Jin X. Y. Zhang H. H. Zhang W. Y. (2017). Baicalein attenuates vinorelbine-induced vascular endothelial cell injury and chemotherapeutic phlebitis in rabbits. Toxicol. Appl. Pharmacol. 318, 23–32. 10.1016/j.taap.2017.01.013 28126410

[B12] Greenberger J. S. (2009). Radioprotection. In Vivo 23, 323–336. 19414422 PMC2981866

[B13] Grémy O. Benderitter M. Linard C. (2008). Acute and persisting Th2-like immune response after fractionated colorectal gamma-irradiation. World J. Gastroenterol. 14, 7075–7085. 10.3748/wjg.14.7075 19084914 PMC2776837

[B14] Guo Z. Hu X. Xing Z. Xing R. Lv R. Cheng X. (2015). Baicalein inhibits prostate cancer cell growth and metastasis *via the* caveolin-1/AKT/mTOR pathway. Mol. Cell Biochem. 406, 111–119. 10.1007/s11010-015-2429-8 25957503 PMC4502300

[B15] Hallahan D. E. Virudachalam S. (1997). Intercellular adhesion molecule 1 knockout abrogates radiation induced pulmonary inflammation. Proc. Natl. Acad. Sci. U. S. A. 94, 6432–6437. 10.1073/pnas.94.12.6432 9177235 PMC21067

[B16] Han S. H. Shim S. Kim M. J. Shin H. Y. Jang W. S. Lee S. J. (2017). Long-term culture-induced phenotypic difference and efficient cryopreservation of small intestinal organoids by treatment timing of Rho kinase inhibitor. World J. Gastroenterol. 23, 964–975. 10.3748/wjg.v23.i6.964 28246470 PMC5311106

[B17] Hatoum O. A. Binion D. G. Otterson M. F. Gutterman D. D. (2003). Acquired microvascular dysfunction in inflammatory bowel disease: loss of nitric oxide-mediated vasodilation. Gastroenterology. 125, 58–69. 10.1016/S0016-5085(03)00699-1 12851871

[B18] Hauer-Jensen M. Denham J. W. Andreyev H. J. (2014). Radiation enteropathy-pathogenesis, treatment and prevention. Nat. Rev. Gastroenterol. Hepatol. 11, 470–479. 10.1038/nrgastro.2014.46 24686268 PMC4346191

[B19] Haydont V. Vozenin-Brotons M. C. (2007). Maintenance of radiation-induced intestinal fibrosis: cellular and molecular features. World J. Gastroenterol. 13, 2675–2683. 10.3748/wjg.v13.i19.2675 17569135 PMC4147115

[B20] Holler V. Buard V. Gaugler M. H. Guipaud O. Baudelin C. Sache A. (2009). Pravastatin limits radiation-induced vascular dysfunction in the skin. J. Invest. Dermatol. 129, 1280–1291. 10.1038/jid.2008.360 19212344

[B21] Jang H. Park S. Lee J. Myung J. K. Jang W. S. Lee S. J. (2018). Rebamipide alleviates radiation-induced colitis through improvement of goblet cell differentiation in mice. J. Gastroenterol. Hepatol. 33, 878–886. 10.1111/jgh.14021 29047150

[B22] Jeong Y. J. Jung M. G. Son Y. Jang J. H. Lee Y. J. Kim S. H. (2015). Coniferyl aldehyde attenuates radiation enteropathy by inhibiting cell death and promoting endothelial cell function. PLoS One. 10, e0128552. 10.1371/journal.pone.0128552 26029925 PMC4452689

[B23] Kavanagh B. D. Pan C. C. Dawson L. A. Das S. K. Li X. A. Ten Haken R. K. (2010). Radiation dose-volume effects in the stomach and small bowel. Int. J. Radiat. Oncol. Biol. Phys. 76, S101–S107. 10.1016/j.ijrobp.2009.05.071 20171503

[B24] Kim D. H. Sung B. Chung H. Y. Kim N. D. (2014). Modulation of colitis-associated colon tumorigenesis by baicalein and betaine. J. Cancer Prev. 19, 153–160. 10.15430/JCP.2014.19.3.153 25337584 PMC4189507

[B25] Korpela E. Liu S. K. (2014). Endothelial perturbations and therapeutic strategies in normal tissue radiation damage. Radiat. Oncol. 9, 266. 10.1186/s13014-014-0266-7 25518850 PMC4279961

[B26] Kwak S. Ku S. K. Han M. S. Bae J. S. (2014). Vascular barrier protective effects of baicalin, baicalein and wogonin *in vitro* and *in vivo*. Toxicol. Appl. Pharmacol. 281, 30–38. 10.1016/j.taap.2014.09.003 25223693

[B27] Lawrence M. B. Springer T. A. (1991). Leukocytes roll on a selectin at physiologic flow rates: distinction from and prerequisite for adhesion through integrins. Cellular 65, 859–873. 10.1016/0092-8674(91)90393-D 1710173

[B28] Lee E. K. Kim J. M. Choi J. Jung K. J. Kim D. H. Chung S. W. (2011). Modulation of NF-κB and FOXOs by baicalein attenuates the radiation-induced inflammatory process in mouse kidney. Free Radic. Res. 45, 507–517. 10.3109/10715762.2011.555479 21284490

[B29] Liu W. Ding I. Chen K. Olschowka J. Xu J. Hu D. (2006). Interleukin 1beta (IL1B) signaling is a critical component of radiation-induced skin fibrosis. Radiat. Res. 165, 181–191. 10.1667/RR3478.1 16435917

[B30] Mabalirajan U. Ahmad T. Rehman R. Leishangthem G. D. Dinda A. K. Agrawal A. (2013). Baicalein reduces airway injury in allergen and IL-13 induced airway inflammation. PLoS One. 8, e62916. 10.1371/journal.pone.0062916 23646158 PMC3639905

[B31] Maj J. G. Paris F. Haimovitz-Friedman A. Venkatraman E. Kolesnick R. Fuks Z. (2003). Microvascular function regulates intestinal crypt response to radiation. Cancer Res. 63, 4338–4341. 12907601

[B32] Nishimoto N. Kishimoto T. (2006). Interleukin 6: from bench to bedside. Nat. Clin. Pract. Rheumatol. 2, 619–626. 10.1038/ncprheum0338 17075601

[B33] Nunes T. Bernardazzi C. de Souza H. S. (2014). Interleukin-33 and inflammatory bowel diseases: lessons from human studies. Mediators Inflamm. 2014, 423957. 10.1155/2014/423957 24701033 PMC3950548

[B34] Okunieff P. Mester M. Wang J. Maddox T. Gong X. Tang D. (1998). In vivo radioprotective effects of angiogenic growth factors on the small bowel of C3H mice. Radiat. Res. 150, 204–211. 10.2307/3579856 9692366

[B35] Paris F. Fuks Z. Kang A. Capodieci P. Juan G. Ehleiter D. (2001). Endothelial apoptosis as the primary lesion initiating intestinal radiation damage in mice. Science. 293, 293–297. 10.1126/science.1060191 11452123

[B36] Peng C. Y. Pan S. L. Huang Y. W. Guh J. H. Chang Y. L. Teng C. M. (2008). Baicalein attenuates intimal hyperplasia after rat carotid balloon injury through arresting cell-cycle progression and inhibiting ERK, Akt, and NF-kappaB activity in vascular smooth-muscle cells. Naunyn Schmiedebergs Arch. Pharmacol. 378, 579–588. 10.1007/s00210-008-0328-1 18663431

[B37] Quarmby S. Kumar P. Kumar S. (1999). Radiation-induced normal tissue injury: role of adhesion molecules in leukocyte-endothelial cell interactions. Int. J. Cancer. 82, 385–395. 10.1002/(SICI)1097-0215(19990730)82:3<385::AID-IJC12>3.0.CO;2-5 10399956

[B38] Radnai B. Sturm E. M. Stančić A. Jandl K. Labocha S. Ferreiros N. (2016). Eosinophils contribute to intestinal inflammation *via* chemoattractant receptor-homologous molecule expressed on Th2 cells, CRTH2, in experimental Crohn’s disease. J. Crohns Colitis. 10, 1087–1095. 10.1093/ecco-jcc/jjw061 26928963 PMC4892354

[B39] Rankin A. L. Mumm J. B. Murphy E. Turner S. Yu N. McClanahan T. K. (2010). IL-33 induces IL-13-dependent cutaneous fibrosis. J. Immunol. 184, 1526–1535. 10.4049/jimmunol.0903306 20042577

[B40] Sato T. Clevers H. (2013). Growing self-organizing mini-guts from a single intestinal stem cell: mechanism and applications. Science 340, 1190–1194. 10.1126/science.1234852 23744940

[B41] Shim S. Lee J. G. Bae C. H. Lee S. B. Jang W. S. Lee S. J. (2015). Claudin-3 expression in radiation-exposed rat models: a potential marker for radiation-induced intestinal barrier failure. Biochem. Biophys. Res. Commun. 456, 351–354. 10.1016/j.bbrc.2014.11.084 25475725

[B42] Shukla P. K. Gangwar R. Manda B. Meena A. S. Yadav N. Szabo E. (2016). Rapid disruption of intestinal epithelial tight junction and barrier dysfunction by ionizing radiation in mouse colon *in vivo*: protection by N-acetyl-l-cysteine. Am. J. Physiol. Gastrointest. Liver Physiol. 310, G705–G715. 10.1152/ajpgi.00314.2015 26822914 PMC4867328

[B43] Takemura N. Kurashima Y. Mori Y. Okada K. Ogino T. Osawa H. (2018). Eosinophil depletion suppresses radiation-induced small intestinal fibrosis. Sci. Transl. Med. 10, eaan0333. 10.1126/scitranslmed.aan0333 29467297

[B44] Toullec A. Buard V. Rannou E. Tarlet G. Guipaud O. Robine S. (2017). HIF-1α deletion in the endothelium, but not in the epithelium, protects from radiation-induced enteritis. Cell. Mol. Gastroenterol. Hepatol. 5, 15–30. 10.1016/j.jcmgh.2017.08.001 29276749 PMC5738457

[B45] Turner J. R. (2009). Intestinal mucosal barrier function in health and disease. Nat. Rev. Immunol. 9, 799–809. 10.1038/nri2653 19855405

[B46] Williams M. R. Azcutia V. Newton G. Alcaide P. Luscinskas F. W. (2011). Emerging mechanisms of neutrophil recruitment across endothelium. Trends Immunol. 32, 461–469. 10.1016/j.it.2011.06.009 21839681 PMC3185121

[B47] Yu J. (2013). Intestinal stem cell injury and protection during cancer therapy. Transl. Cancer Res. 2, 384–396. 10.3978/j.issn.2218-676X.2013.07.03 24683536 PMC3966653

